# Supplementary figures and images for: Onion bulb extract can both reverse and prevent colitis in mice via inhibition of pro-inflammatory signaling molecules and neutrophil activity
Source: PLoS One. 2020 Oct 23;15(10):e0233938. doi: 10.1371/journal.pone.0233938 (PMC7584208; doi:10.1371/journal.pone.0233938)

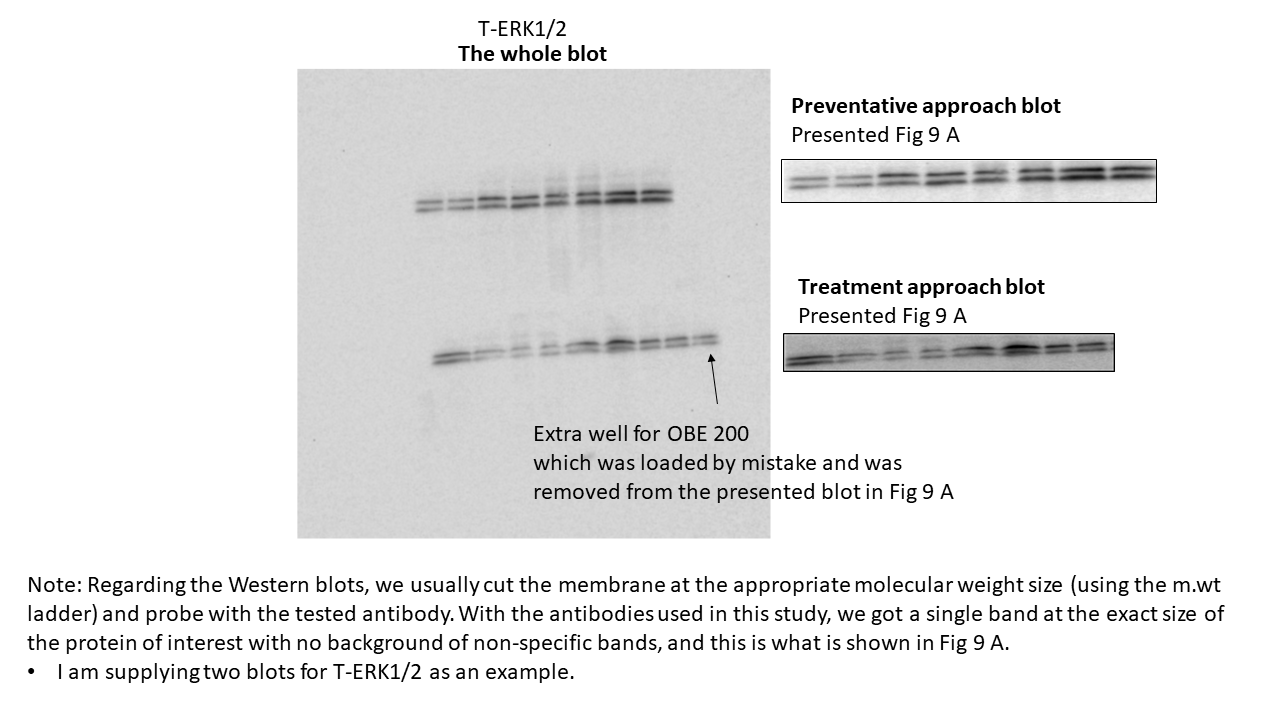

Supplement: S1 Fig — (TIF) [file pone.0233938.s001.tif]
