# Supplementary material for: Onion bulb extract can both reverse and prevent colitis in mice via inhibition of pro-inflammatory signaling molecules and neutrophil activity
Source: PLoS One. 2020 Oct 23;15(10):e0233938. doi: 10.1371/journal.pone.0233938 (PMC7584208; doi:10.1371/journal.pone.0233938)
Supplement: S1 Raw data — (DOCX) [file pone.0233938.s002.docx]

**Raw data**

**weight change, preventative approach**

| UT | | Vehicle | | 100mg/kg OE | | 200mg/kg OE | |
| --- | --- | --- | --- | --- | --- | --- | --- |
| 100.0 | 0.0 | 100.0 | 0.0 | 100.0 | 0.0 | 100.0 | 0.4 |
| 101.0 | 1.0 | 96.0 | 2.6 | 95.0 | 3.0 | 99.9 | 0.4 |
| 102.0 | 1.5 | 97.0 | 2.8 | 94.0 | 4.0 | 99.5 | 0.5 |
| 101.0 | 2.0 | 97.0 | 2.8 | 96.0 | 2.6 | 99.4 | 0.5 |
| 103.0 | 3.0 | 97.6 | 2.8 | 93.0 | 4.7 | 98.9 | 0.5 |
| 102.0 | 2.0 | 98.0 | 2.6 | 93.0 | 4.8 | 98.4 | 0.5 |
| 102.0 | 1.0 | 100.0 | 3.0 | 95.0 | 4.0 | 98.3 | 0.4 |
| 102.0 | 1.0 | 103.0 | 3.0 | 100.0 | 4.0 | 97.6 | 0.4 |
| 102.0 | 1.0 | 104.0 | 4.0 | 98.0 | 4.0 | 97.6 | 0.4 |
| 102.0 | 1.0 | 107.0 | 3.0 | 99.0 | 4.0 | 97.6 | 0.4 |
| 102.0 | 1.0 | 105.0 | 2.0 | 104.0 | 4.0 | 97.6 | 0.5 |
| 102.0 | 1.0 | 104.0 | 2.0 | 104.0 | 4.0 | 97.6 | 0.5 |
| 102.0 | 1.0 | 101.0 | 4.0 | 99.0 | 3.0 | 97.5 | 0.5 |
| 102.0 | 1.0 | 92.0 | 5.0 | 92.0 | 6.0 | 97.5 | 0.5 |

mean SEM mean SEM mean SEM mean SEM

**colon length, preventative approach**

| UT | | Vehicle | | 100mg/kg | | 200mg/kg | |
| --- | --- | --- | --- | --- | --- | --- | --- |
| 82. | 5.8 | 66.0 | 4.0 | 72.0 | 4.0 | 75. | 2.0 |

mean SEM mean SEM mean SEM mean SEM

**colon thickness, preventative approach**

| UT | | Vehicle | | 100mg/kg | | 200mg/kg | |
| --- | --- | --- | --- | --- | --- | --- | --- |
| 0.47 | 0.03 | 0.62 | 0.05 | 0.53 | 0.04 | 0.55 | 0.02 |

mean SEM mean SEM mean SEM mean SEM

**histological score of colitis, preventative approach**

| UT | | Vehicle | | 100mg/kg | | 200mg/kg | |
| --- | --- | --- | --- | --- | --- | --- | --- |
| 0.1 | 0.001. | 9.60 | 0.40 | 7. | 0.50 | 5.0 | 0.6 |

mean SEM mean SEM mean SEM mean SEM

**% ulceration, preventative approach**

| UT | | Vehicle | | 100mg/kg | | 200mg/kg | |
| --- | --- | --- | --- | --- | --- | --- | --- |
| 1.0 | 0.001 | 67.0 | 5.7 | 49.0 | 4.7 | 28. | 3.5 |

|  |  |  |  |  |  |
| --- | --- | --- | --- | --- | --- |

mean SEM mean SEM mean SEM mean SEM

**IF, COX2, preventative approach**

| UT | Vehicle | OBE 100mg/kg | OBE 200mg/kg |
| --- | --- | --- | --- |
| 3.320 | 9.068 | 3.550 | 3. |
| 2.748 | 7.560 | 5.051 | 3. |
| 3.323 | 7.162 | 3.361 | 4. |
| 2.480 | 11.775 | 3.542 | 1. |
| 1.544 | 7.669 | 5.221 | 1. |
| 3.091 | 9.760 | 3.186 |  |
| 3.897 |  | 2.877 |  |
| 3.457 |  | 1.932 |  |
|  |  | 1.740 |  |

**IF, P-AKT, preventative approach**

| UT | Vehicle | OBE 100mg/kg | OBE 200mg/kg |
| --- | --- | --- | --- |
| 2.981 | 16.431 | 5.736 | 3. |
| 3.458 | 8.576 | 11.530 | 4. |
| 2.643 | 13.117 | 6.336 | 5. |
| 3.399 | 11.523 | 4.529 | 4. |
| 4.328 | 9.427 | 3.668 |  |
| 4.823 | 10.383 | 5.615 |  |
| 3.154 | 11.636 | 11.798 |  |
|  | 9.132 | 4.420 |  |
|  | 8.975 |  |  |

**IF, P-ERK1/2, preventative approach**

| UT | Vehicle | OBE 100mg/kg | OBE 200mg/kg |
| --- | --- | --- | --- |
| 2.120 | 7.497 | 5.286 | 4. |
| 5.759 | 9.130 | 7.172 | 3. |
| 6.418 | 11.648 | 3.779 | 4. |
| 4.711 | 18.326 | 6.999 | 5. |
| 4.267 | 14.369 | 5.814 | 3. |
|  | 9.320 | 2.925 |  |
|  | 12.376 | 6.559 |  |
|  | 9.664 | 6.705 |  |
|  | 8.768 | 6.987 |  |
|  |  | 5.044 |  |
|  |  | 1.938 |  |
|  |  | 10.920 |  |
|  |  | 12.293 |  |

**IF, P-p38, preventative approach**

| UT | Vehicle | OBE 100mg/kg | OBE 200mg/kg |
| --- | --- | --- | --- |
| 6.334 | 9.206 | 10.558 | 4. |
| 6.426 | 10.472 | 6.430 | 6. |
| 8.288 | 10.962 | 7.618 | 3. |
| 6.917 | 14.211 | 8.203 | 4. |
| 6.264 | 8.903 |  |  |
| 6.171 | 13.513 |  |  |
|  | 12.795 |  |  |
|  |  |  |  |

**weight change, treatment approach**

| UT | | Vehicle | | 100mg/kg OE | | 200mg/kg OE | |
| --- | --- | --- | --- | --- | --- | --- | --- |
| 100.0 | 0.0 | 100.0 | 0.0 | 100.0 | 0.0 | 100.0 | 0.0 |
| 100.0 | 1.0 | 100.0 | 0.5 | 96.0 | 0.5 | 99.7 | 0.2 |
| 99.0 | 1.5 | 97.0 | 0.5 | 96.0 | 0.6 | 99.2 | 0.7 |
| 101.0 | 2.0 | 93.0 | 0.3 | 97.0 | 0.7 | 96.2 | 1.4 |
| 103.0 | 3.0 | 90.0 | 0.5 | 95.0 | 0.7 | 95.5 | 1.0 |
| 102.0 | 2.0 | 85.0 | 0.5 | 94.0 | 0.7 | 91.0 | 1.4 |
| 102.0 | 1.0 | 83.0 | 1.0 | 94.0 | 1.2 | 93.0 | 1.3 |
|  |  |  |  |  |  |  |  |
|  |  |  |  |  |  |  |  |

mean SEM mean SEM mean SEM mean SEM

**colon length, treatment approach**

| UT | | Vehicle | | 100 OBE | | 200 OBE | |
| --- | --- | --- | --- | --- | --- | --- | --- |
| 82. | 5.8 | 66. | 2.5 | 72.0 | 2.0 | 80. | 2.0 |

mean SEM mean SEM mean SEM mean SEM

**colon thickness, treatment approach**

| UT | | Vehicle | | 100 OBE | | 200 OBE | |
| --- | --- | --- | --- | --- | --- | --- | --- |
| 0.50 | 0.04 | 0.61 | 0.02 | 0.50 | 0.03 | 0.40 | 0.03 |

mean SEM mean SEM mean SEM mean SEM

**histological score of colitis, treatment approach**

| UT | | Vehicle | | 100mg/kg | | 200mg/kg | |
| --- | --- | --- | --- | --- | --- | --- | --- |
| 0.1 | 0. | 10.30 | 0.27 | 8.4 | 0.45 | 8.5 | 0.65 |

mean SEM mean SEM mean SEM mean SEM

**% ulceration, treatment approach**

| UT | | Vehicle | | 100mg/kg | | 200mg/kg | |
| --- | --- | --- | --- | --- | --- | --- | --- |
| 1.0 |  | 58.0 | 5.6 | 46.0 | 4.3 | 46. | 6. |

mean SEM mean SEM mean SEM mean SEM

**IF, COX2, treatment approach**

| UT | Vehicle | 100mg/kg | 200mg/kg |
| --- | --- | --- | --- |
| 3.320 | 10.389 | 8.364 | 4.968 |
| 2.748 | 8.947 | 3.665 | 5.646 |
| 3.323 | 8.242 | 3.155 | 5.299 |
| 2.480 | 10.847 | 3.367 | 3.161 |
| 1.544 | 9.686 | 6.014 | 5.570 |
| 3.091 | 11.775 | 4.292 | 4.249 |
| 3.897 |  | 4.270 | 1.913 |
| 3.457 |  | 5.122 | 2.144 |
|  |  |  | 1.417 |
|  |  |  |  |

**IF, P-AKT, treatment approach**

| UT | Vehicle | 100mg/kg | 200mg/kg |
| --- | --- | --- | --- |
| 2.981 | 8.233 | 8.125 | 9.014 |
| 3.458 | 13.786 | 4.281 | 4.356 |
| 2.643 | 17.657 | 9.579 | 9.585 |
| 3.399 | 15.416 | 7.313 | 10.166 |
| 4.328 | 19.651 | 7.899 | 12.206 |
| 4.823 | 16.270 | 7.931 | 1.778 |
| 3.154 | 22.427 | 11.423 | 7.546 |
|  | 19.334 | 15.672 | 9.310 |
|  | 16.008 | 11.451 | 9.966 |
|  |  | 13.482 | 13.350 |
|  |  |  | 5.776 |

**IF, P-ERK1/2, treatment approach**

| UT | Vehicle | 100mg/kg | 200mg/kg |
| --- | --- | --- | --- |
| 2.120 | 14.205 | 8.201 | 2.742 |
| 5.759 | 15.923 | 9.508 | 5.910 |
| 6.418 | 12.530 | 11.026 | 7.280 |
| 4.711 | 10.190 | 11.526 | 8.052 |
| 4.267 | 12.512 | 10.015 | 1.927 |
| 4.267 | 9.717 | 11.610 | 1.927 |
|  | 7.258 | 1.928 | 9.933 |
|  | 21.128 | 8.246 | 4.052 |
|  | 11.701 | 8.451 | 8.047 |
|  | 9.457 | 11.230 | 7.202 |
|  | 9.912 | 9.666 |  |
|  | 13.731 |  |  |
|  | 15.820 |  |  |
|  |  |  |  |

**IF, P-p38, treatment approach**

| UT | Vehicle | 100mg/kg | 200mg/kg |
| --- | --- | --- | --- |
| 6.334 | 11.199 | 6.561 | 9.549 |
| 6.426 | 10.255 | 9.460 | 9.295 |
| 8.288 | 10.474 | 9.184 | 7.082 |
| 6.917 | 7.244 | 7.710 | 6.470 |
| 6.264 | 8.146 | 8.549 | 6.476 |
| 6.171 | 9.759 | 9.127 | 9.394 |
|  | 10.620 | 8.672 | 6.365 |
|  | 10.550 | 8.236 | 7.445 |
|  |  |  |  |

**% viable neutrophil**

| Vehicle | | 10ng/ml | | 100ng/ml | | 1μg/ml | | 10μg/ml | | 100μg/ml | |
| --- | --- | --- | --- | --- | --- | --- | --- | --- | --- | --- | --- |
| 60.00 | 4.20 | 71.00 | 2.40 | 72.20 | 3.00 | 32.04 | 2.50 | 24.90 | 4.80 | 2.10 | 0.10 |

**% apoptotic neutrophils**

| Vehicle | | 10ng/ml | | 100ng/ml | | 1μg/ml | | 10μg/ml | | 100μg/ml | |
| --- | --- | --- | --- | --- | --- | --- | --- | --- | --- | --- | --- |
| 28.0 | 3.1 | 17.3 | 2.7 | 19.00 | 2.00 | 56.91 | 3.10 | 52.6 | 4.2 | 89.8 | 1.9 |

**Superoxide release**

**neutrophils alone**

**mean SEM**

| 599.666700 | 107.611200 |
| --- | --- |
| 2177.000000 | 646.145900 |
| 5674.667000 | 750.595200 |
| 8477.333000 | 777.799900 |
| 11225.000000 | 777.659300 |
| 15802.000000 | 583.381500 |
| 20591.330000 | 980.840800 |
| 23478.670000 | 1841.310000 |
| 23656.000000 | 1788.895000 |
| 21922.000000 | 1530.832000 |
| 19518.000000 | 1618.156000 |
| 16947.000000 | 2274.368000 |
| 13902.000000 | 2658.647000 |
| 10771.330000 | 2229.725000 |
| 8575.333000 | 1368.323000 |
| 7452.333000 | 1084.894000 |
| 6841.000000 | 748.534900 |
| 6496.333000 | 716.170500 |
| 6120.667000 | 600.516600 |
| 5862.667000 | 551.391100 |
| 5835.667000 | 387.098400 |
| 5775.667000 | 420.952700 |
| 5657.333000 | 322.292800 |
| 5527.667000 | 400.647200 |
| 5397.667000 | 433.555300 |
| 5300.000000 | 347.111700 |
| 5327.667000 | 331.047800 |
| 5195.000000 | 378.999300 |
| 5328.333000 | 372.798400 |
| 5292.667000 | 460.628000 |
|  |  |
|  |  |

**neutrophils plus WKYMVm**

**mean SEM**

| 19215.330000 | 4483.025000 |
| --- | --- |
| 132270.700000 | 8558.605000 |
| 230325.300000 | 5800.271000 |
| 219520.700000 | 14242.500000 |
| 317947.700000 | 45185.210000 |
| 481121.300000 | 67838.150000 |
| 601041.000000 | 90580.450000 |
| 674183.700000 | 118915.400000 |
| 746138.300000 | 146936.200000 |
| 817538.000000 | 163483.800000 |
| 857119.300000 | 164508.500000 |
| 863481.000000 | 154394.200000 |
| 855210.000000 | 141206.000000 |
| 859989.700000 | 133093.400000 |
| 895561.700000 | 123288.300000 |
| 953419.000000 | 101476.000000 |
| 1005150.000000 | 72223.170000 |
| 1020688.000000 | 60367.570000 |
| 983345.000000 | 71901.520000 |
| 893882.000000 | 83157.640000 |
| 782428.000000 | 89867.860000 |
| 654307.300000 | 90050.840000 |
| 529776.000000 | 80303.630000 |
| 421894.300000 | 65191.130000 |
| 340316.000000 | 52364.150000 |
| 283663.000000 | 40952.320000 |
| 248690.000000 | 35392.990000 |
| 230749.000000 | 32710.480000 |
| 223170.700000 | 32622.910000 |
| 223361.300000 | 34796.000000 |

**neutrophils plus WKYMVm plus OBE 10 ng/ml**

**mean SEM**

| 25928.000000 | 8945.730000 |
| --- | --- |
| 124012.300000 | 21518.440000 |
| 172099.000000 | 9366.488000 |
| 159291.000000 | 17567.790000 |
| 234063.000000 | 36552.580000 |
| 334640.300000 | 42961.460000 |
| 407015.700000 | 45194.730000 |
| 464700.300000 | 52336.450000 |
| 530978.700000 | 66833.570000 |
| 599730.700000 | 76855.910000 |
| 657119.300000 | 164508.500000 |
| 670119.000000 | 89474.580000 |
| 665995.300000 | 99255.010000 |
| 653645.300000 | 113508.100000 |
| 651442.300000 | 127204.100000 |
| 656493.700000 | 138788.300000 |
| 654372.700000 | 147161.900000 |
| 631203.700000 | 147559.800000 |
| 583459.300000 | 138584.800000 |
| 518560.300000 | 123297.400000 |
| 444685.000000 | 103976.900000 |
| 365460.000000 | 84005.860000 |
| 289783.300000 | 63425.570000 |
| 225935.300000 | 44949.320000 |
| 177857.000000 | 31868.630000 |
| 142347.700000 | 23509.210000 |
| 116640.000000 | 17879.320000 |
| 99159.340000 | 16037.340000 |
| 87000.000000 | 14434.340000 |
| 77756.000000 | 13596.280000 |

**neutrophils plus WKYMVm plus OBE 100 ng/ml**

**mean SEM**

| 170939.000000 | 24333.580000 |
| --- | --- |
| 325669.700000 | 20320.430000 |
| 274850.700000 | 17787.440000 |
| 367192.300000 | 51364.360000 |
| 372269.300000 | 58911.930000 |
| 307740.300000 | 55162.250000 |
| 417683.300000 | 47154.830000 |
| 347560.300000 | 40269.830000 |
| 287536.700000 | 37950.130000 |
| 226407.700000 | 33046.720000 |
| 175412.300000 | 29343.000000 |
| 144548.700000 | 29608.600000 |
| 140408.700000 | 34145.250000 |
| 161932.300000 | 37954.730000 |
| 202510.300000 | 36518.770000 |
| 241625.300000 | 30374.580000 |
| 257451.300000 | 27336.310000 |
| 242821.000000 | 27853.460000 |
| 208004.000000 | 27633.610000 |
| 168976.300000 | 24694.270000 |
| 131545.000000 | 19693.760000 |
| 103101.000000 | 15727.690000 |
| 81029.000000 | 11956.470000 |
| 64874.000000 | 9932.694000 |
| 54759.670000 | 10022.250000 |
| 48084.670000 | 10955.230000 |
| 45222.670000 | 12505.030000 |
| 44196.670000 | 13396.880000 |
| 44577.670000 | 13849.320000 |

**neutrophils plus WKYMVm plus OBE 1 ug/ml**

**mean SEM**

| 4706.333000 | 872.052900 |
| --- | --- |
| 40556.000000 | 3362.207000 |
| 77158.000000 | 3825.108000 |
| 71947.660000 | 3262.262000 |
| 94137.340000 | 11198.350000 |
| 132634.700000 | 16314.620000 |
| 151108.000000 | 20504.880000 |
| 154203.000000 | 24902.750000 |
| 159969.700000 | 29587.400000 |
| 166391.300000 | 32345.610000 |
| 167604.700000 | 32758.690000 |
| 164026.000000 | 31329.140000 |
| 160456.300000 | 29781.340000 |
| 161395.000000 | 29061.590000 |
| 170775.300000 | 27987.980000 |
| 187041.000000 | 26567.730000 |
| 201066.000000 | 25823.170000 |
| 207182.000000 | 26362.530000 |
| 200706.000000 | 28747.410000 |
| 182006.000000 | 28785.430000 |
| 158778.000000 | 28147.080000 |
| 132391.300000 | 25488.720000 |
| 107703.700000 | 21855.610000 |
| 86133.660000 | 17544.520000 |
| 70221.000000 | 14118.070000 |
| 59241.330000 | 11284.850000 |
| 52354.670000 | 9568.856000 |
| 49210.670000 | 8917.685000 |
| 48258.670000 | 8802.596000 |
| 48966.000000 | 9286.016000 |
|  |  |

**neutrophils plus WKYMVm plus OBE 10 ug/ml**

**mean SEM**

| 3435.333000 | 880.628800 |
| --- | --- |
| 14431.330000 | 2385.609000 |
| 26451.330000 | 1596.554000 |
| 32026.000000 | 2072.293000 |
| 39559.000000 | 2555.523000 |
| 44644.330000 | 2598.692000 |
| 45705.330000 | 2161.726000 |
| 46006.670000 | 2120.080000 |
| 48003.330000 | 3362.046000 |
| 50768.670000 | 3882.056000 |
| 53851.000000 | 4597.955000 |
| 55067.670000 | 5177.770000 |
| 54010.330000 | 5688.229000 |
| 52940.670000 | 7064.513000 |
| 51492.330000 | 7740.098000 |
| 51059.670000 | 8586.957000 |
| 49917.330000 | 9367.990000 |
| 47739.670000 | 9434.567000 |
| 44046.330000 | 9051.880000 |
| 39805.000000 | 8238.156000 |
| 35013.000000 | 7232.661000 |
| 29530.670000 | 6065.424000 |
| 24565.000000 | 4646.794000 |
| 20149.330000 | 3677.309000 |
| 16770.000000 | 2759.122000 |
| 14224.670000 | 2210.442000 |
| 11886.000000 | 1779.969000 |
| 10515.000000 | 1657.219000 |
| 9353.333000 | 1487.907000 |
| 8272.333000 | 1321.848000 |

**neutrophils plus WKYMVm plus OBE 100 ug/ml**

**mean SEM**

| 2258.667000 | 818.846900 |
| --- | --- |
| 39355.000000 | 6613.856000 |
| 73928.340000 | 5086.650000 |
| 61364.330000 | 4118.225000 |
| 80982.660000 | 11951.020000 |
| 113084.300000 | 14497.450000 |
| 112134.000000 | 13022.510000 |
| 91111.340000 | 10887.000000 |
| 75210.340000 | 8901.892000 |
| 62445.000000 | 8300.690000 |
| 48767.670000 | 7019.339000 |
| 37815.670000 | 5888.347000 |
| 31282.670000 | 6299.720000 |
| 30515.670000 | 6929.469000 |
| 35322.000000 | 7609.330000 |
| 44716.670000 | 7815.592000 |
| 53505.670000 | 6676.072000 |
| 57177.330000 | 6123.349000 |
| 53964.670000 | 6476.958000 |
| 46537.000000 | 5874.401000 |
| 38065.330000 | 5133.126000 |
| 29521.000000 | 4067.331000 |
| 22744.000000 | 3081.037000 |
| 18072.670000 | 2344.813000 |
| 14470.330000 | 1727.545000 |
| 12324.330000 | 1692.130000 |
| 10834.330000 | 1930.530000 |
| 4967.000000 | 2618.840000 |
| 9814.667000 | 2394.328000 |
| 9991.667000 | 2446.075000 |
|  |  |
