# Supplementary material for: Onion bulb extract can both reverse and prevent colitis in mice via inhibition of pro-inflammatory signaling molecules and neutrophil activity
Source: PLoS One. 2020 Oct 23;15(10):e0233938. doi: 10.1371/journal.pone.0233938 (PMC7584208; doi:10.1371/journal.pone.0233938)
Supplement: Attachment — Submitted filename: Response to reviewers.docx [file pone.0233938.s003.docx]

**Response to reviewers’ comments**

First, we would like to thank the reviewers for their valuable comments to increase the quality of this manuscript.

**Reviewer #1:**

In the current study, authors investigated the impact of Onion bulb extract (OBE) on colitis severity in BALB/c mice models. Here are my concerns:
1- The methodology was not well described; the extraction procedure, macroscopic assessment of colitis severity, immunofluorescence, and isolation of bone-marrow derived neutrophils methods should be well explained.

Response: a detailed methodology for the above mentioned procedures is now included in the revised version of the manuscript. We also performed western blotting analysis for the expression/activity of various signaling molecules and immune cell markers as suggested by the reviewers, and therefore we included a methodology section for this technique in the revised version of the manuscript.

2- Estimation of the used OBE doses should be explained.

Response: the following sentence is now included in the (**result)** section:

*We choose 100-200 mg/kg dose of OBE based on our previously published report using the prophylactic approach [25] which demonstrated anti-inflammatory properties using the same colitis model.*

3- Why they used IP injection instead of oral administration?

Response: we tried oral gavage and coating the food with OBE but we did not observe any anti-inflammatory properties of OBE (unpublished data). Therefore, we used the i.p route and clearly showed anti-inflammatory properties using various approaches; prophylactic (previous publication, Ref 25), and preventative and treatment (current manuscript).

4- It is not cleared they used an alcoholic OBE extract or aqueous OBE extract.

Response: dichloromethane was used as the extraction solvent. This is now clear after adding the detailed extraction procedure to the revised version of the manuscript.

The following paragraph is now included in the discussion section of the revised version of the manuscript:

*Most of the documented anti-inflammatory activities shown by onion are attributed to the known flavonoid quercetin, where polar solvents, particularly water and/or alcohols, were used to prepare the tested extracts. Therefore, in order to investigate the possibility of having other ingredients with promising activities, particularly in reducing the colitis severity, the applied extraction method in this study was selected to provide extracts with, mainly, non-polar constituents, which, in a way, resembles the onion essential oils, where the highly polar bioactive flavonoids, including quercetin, are not among them. This conclusion was further confirmed through the chromatographic analysis of the extracts. Thus, dichloromethane was selected, as the extraction solvent, over water or alcohols. Hence, the observed promising activities of the extract in reducing the colitis severity can be attributed to the non-polar constituents. These fat-soluble constituents are likely to be the sulfur-containing compounds which are found excessively in the essential oil of onion.*

5- Herbs extracts contain various active ingredients like flavonoids and etc., the authors should be specified that which compound is responsible for the observed modulatory results in their investigation.

Response: It is true that various active ingredients do present in herb extracts, however, most of the documented anti-inflammatory activities shown by onion are attributed to the known flavonoid quercetin, where polar solvents, particularly water and/or alcohols, were used to prepare the tested extracts. However, to fulfill this study aim; to investigate the possibility of having other ingredients with promising activities, particularly in reducing the colitis severity, the applied extraction method (using the nonpolar solvent, dichloromethane) in this study was selected to provide extracts with, mainly, non-polar constituents, which, in a way, resembles the onion essential oils, where the highly polar bioactive flavonoids, including quercetin, are not among them. Hence, the observed promising activities of the extract in reducing the colitis severity can be attributed to the non-polar constituents. These fat-soluble constituents are likely to be the sulfur-containing compounds which are found excessively in the essential oil of onion. Our ultimate goal is to unambiguously identify the active ingredient(s) in this extract, however, this is a whole different project and is beyond the scope of our current study.

The above paragraph was added to the discussion section to justify the use of dichloromethane over water and/or alcohols.

6- The results of OBE 200 mg/kg impact on pro-inflammatory molecules are not shown in the Figures 7 & 8 for preventative approach.

Response: we now included IF analysis for the various tested pro-inflammatory molecules using the 200 mg/kg dose for the preventative approach in the revised version of the manuscript (NEW Figs 7 and 8). We also included western blotting analysis of the tested molecules using 100 and 200 mg/kg doses of OBE for the preventative and treatment approaches in the revised version of the manuscript (NEW Fig 9 A).
7- Sample size estimation is not clarified. The number of mice are varied between examined groups and may impact on the overall conclusion. Why we had more animal subjects (almost double) in 100 OBE mg/kg group than the 200 OBE mg/kg group?

Response: we included large number of mice in all groups and we were including the 100 mg/kg dose as (*positive control)* in each experiment (based on our previously published report using the prophylactic approach, Ref 25). In general, there is minute variability between individual mice in each group, which is confirmed by tight range of SEM in the figures.

8- For a better conclusion, Comparative analysis between two different OBE doses in all of the examinations is needed.

Response: we now included IF analysis for the various tested pro-inflammatory molecules using the 200 mg/kg dose for the preventative approach in the revised version of the manuscript (NEW Figs 7 and 8). We also included western blotting analysis of the tested molecules using 100 and 200 mg/kg doses of OBE for the preventative and treatment approaches in the revised version of the manuscript (NEW Fig 9 A).

A comparative analysis is now included for these two doses of OBE using IF and western blotting analysis. There is no further enhancement in the anti-inflammatory effects of OBE using the 200 dose when compared to the 100 mg/kg dose.

**Reviewer #2:**

The manuscript by Dr. Maitham Khajah et al., suggests that Onion bulb extract (OBE) can reverse and prevent colitis in mice via inhibition of pro-inflammatory signaling molecules and neutrophil activity. The authors demonstrated that OBE treatment significantly reduced colitis severity, the colonic expression/activity profile of pro-inflammatory molecules, inhibited neutrophils-induced superoxide release, and increased spontaneous apoptosis of neutrophils in vitro. The data demonstrated the anti-inflammatory properties of onion bulb extract (OBE) in reducing colitis severity in mice. The study is well designed and logical but partially supported by the data. The experiments are well developed, need some in-vivo effector site protection of colon as compared to bone marrow-derived induced neutrophils function. The specific comments are:

1. The figure shows the effects of OBE on DSS induced colitis. The changes in colon infiltrating cells is crucial for any effector function is missing. Further, it is not clear that what is the maximum effective dose as 100 or 200 mg/kg body weight.

Response: we are now providing western blotting analysis of the tested pro-inflammatory molecules and immune cell markers (CD3; T-cells, F4/80; macrophages, Ly6G; neutrophils, and CD19; B-cells) for 100 and 200 mg/kg doses of OBE in the revised version of the manuscript (new Figs 7, 8, and 9 A). We showed that the expression level of neutrophils (but not macrophage) marker was significantly increased in DSS-treated mice and was reduced by OBE treatment using the preventative and treatment approaches. Both T- and B-cell markers were not detected by western blotting analysis even when very high concentration of primary or secondary antibodies were used (data not shown).

In regards to the macroscopic assessment, 200 mg/kg was more potent in reducing colitis severity parameters when compared to the 100 mg/kg using both approaches (Tables 1 and 2). In regards to the microscopic assessment, there is slight improvement with the 200 mg/kg (only with the preventative but not the treatment approach) when compared with the 100 mg/kg.

In general, 100 mg/kg dose is the maximum effective dose of OBE since no significant additional improvement was observed by the 200 mg/kg dose

2. The percentage of changes in body weight data is very nice. It is not clear as to which immune cells recruited to the colon and OBE treatment reduce/enhances those immune populations.

Response: we are now providing western blotting analysis of the immune cell markers for 100 and 200 mg/kg doses of OBE in the revised version of the manuscript (NEW Fig 9 A). We showed that the expression level of neutrophils (but not macrophage) marker was significantly increased in DSS-treated mice and was reduced by OBE treatment using the preventative and treatment approaches. Both T- and B-cell markers were not detected by western blotting analysis even when very high concentration of primary or secondary antibodies were used (data not shown).

3. Need a strong rationale for using bone marrow-derived neutrophils in place of neutrophils present in the colon during DSS and OBE treatment.

Response: this is a very valid comment, and we choose to use naïve bone-marrow-derived neutrophils rather than neutrophils present at the site of inflammation (colon) for the following reasons: a) to perform the *in vitro* assays we need very large number of cells which can be obtained easily from the bone marrow but not from peripheral organs (e.g. colon) or the blood. b) most immune cells present at inflammatory site are already primed/desensitized due to the presence of large amounts of pro-inflammatory molecules which might affect their function/behavior *ex-vivo*. c) we wanted to test these cells specifically in response to stimulation with a bacterially-derived stimulus WKYMVm (fMLP-peptide) since bacteria/bacterial products are considered one of the main driving force for colitis in humans and various animal models. d) we already used bone-marrow derived neutrophils previously for various *in vitro* assays linked to colitis, please see the below published manuscripts from our group:

PMID: 28487234, PMID: 23355730, PMID: 21393420, PMID: 20872834, PMID: 27997590

4. Histological data at both doses (100 or 200 mg/kb) shows similar results. I suggest PI to just follow one dose for clarity in this manuscript.

Response: we agree with the reviewer comment regarding the similarity in the response obtained between these two doses at the histological level, but we thought to keep both doses since there is significant difference at the macroscopic level (Tables 1 and 2).

5. The observation of changes in COX-2, P-AKT, P ERK1/2 through immunofluorescence analysis is not clear and needs a better technique like western for a prudent conclusion.

Response: we are now providing western blotting analysis of the tested molecules for 100 and 200 mg/kg doses of OBE in the revised version of the manuscript (NEW Fig 9 A).

6. Fig.7 is a very poor quality presentation. It is not clear as cells are making these or a just antibodies background. Need some better techniques like immunocytochemistry (works better in paraffin section).

Response: we are now providing western blotting analysis of the tested molecules which gives better quantification analysis of these proteins (NEW Fig 9 A).
